# From intestinal metabolites to the brain: Investigating the mysteries of Long COVID

**DOI:** 10.1002/ctm2.1608

**Published:** 2024-03-11

**Authors:** Shan Liu, Ashwarya S. Devason, Maayan Levy

**Affiliations:** ^1^ Department of Microbiology University of Pennsylvania School of Medicine Philadelphia Pennsylvania USA

## 1 INTRODUCTION

To date, more than 770 million individuals have been infected with SARS‐CoV‐2 worldwide.^1^ Postacute sequelae of COVID‐19 (PASC), commonly called ‘Long COVID’, constitute a primary public health concern. Long COVID involves symptoms that emerge, persist, or relapse over more than 30 days after acute SARS‐CoV‐2 infection. The symptoms of PASC span a wide range of organs and are heterogeneous among patients. A recent study aimed at developing a comprehensive definition of PASC based on symptoms in a prospective cohort study involving 9764 patients.^2^ Given the intricacies of PASC, the underlying etiology remains poorly understood, posing an urgent global health challenge that necessitates resolution. We have recently conducted a study that may shed new light on one of the possible biological causes of PASC.^3^

This study began with exploring metabolite profiles that are distinct in Long COVID patients. Among the most significantly depleted metabolites observed in acute and postacute patients, compared to recovered patients, was serotonin (5‐hydroxytryptamine, 5‐HT). Mouse models of viral infection and an intestinal organoids model were employed to elucidate the molecular mechanisms underlying this phenotype. We found that viral RNA triggered type I interferon (IFN) signalling, which in turn suppressed serotonin levels by affecting serotonin production, storage and degradation.

Beyond establishing a possible association between 5‐HT and PASC and elucidating the potential molecular mechanisms underlying this connection, our study further suggests that the decrease of peripheral serotonin can lead to impaired hippocampal responses and memory by impeding the activity of the vagus nerve. The findings of this investigation may contribute to our understanding of the mechanisms involved in PASC and suggest new predictive biomarkers and potential therapeutic targets for intervention.

## 2 THE SEROTONIN HYPOTHESIS AND MECHANISMS OF DEPLETION

The reduction of serotonin in plasma was identified through a plasma metabolomics analysis conducted on 58 PASC patients, compared to 30 individuals with symptom‐free recovery and 60 individuals with acute COVID‐19. Consistent with these findings in human cohorts, a reduction in plasma serotonin was observed in the SARS‐CoV‐2‐infected K18‐ACE2 mouse model, which expresses human ACE2. By using synthetic double‐stranded RNA polyinosinic:polycytidylic acid (poly(I:C)) or viral infection, this study additionally revealed that the reduction in serotonin was mediated by the viral‐induced type I interferon response in a TLR3‐ and STAT1‐dependent manner.

**FIGURE 1:**
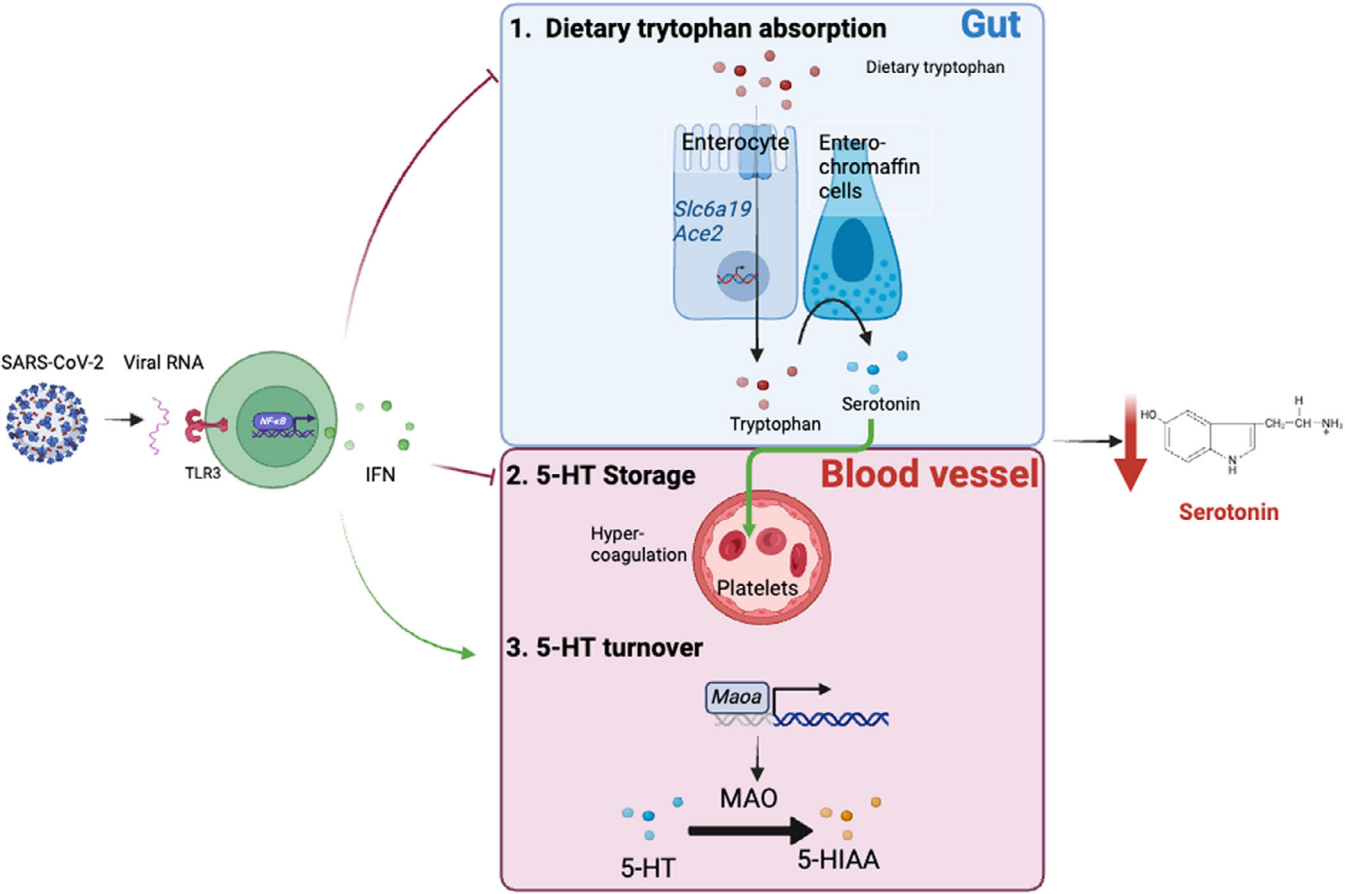
Three distinct mechanisms of serotonin depletion induced by viral inflammation.

We next investigated the mechanisms through which viral‐induced inflammation leads to a reduction in peripheral serotonin levels (Figure 1). Notably, tryptophan, the precursor of serotonin that is used as a substrate for serotonin synthesis by enterochromaffin cells in the gastrointestinal tract, was also diminished in PASC patients. Using a mouse model, organoids and intestinal epithelial cells, we uncovered that viral‐driven inflammation inhibits tryptophan absorption by suppressing the epithelial expression of amino acid uptake genes, such as (*Slc6a19*), which encodes for the neutral amino acid transporter B^0^AT1, and its chaperone ACE2.

In addition to suppression of serotonin production, we observed that viral inflammation also impaired serotonin storage and turnover. Following synthesis in enterochromaffin cells, platelets store and transport peripheral serotonin and MAO enzymes rapidly turn over free serotonin. Notably, platelet counts were decreased following viral infection or poly(I:C) treatment, suggesting that the carrying capacity for serotonin in the systemic circulation was diminished. This reduction could not be rescued by tryptophan supplementation, indicating that the impaired storage was independent of reduced amino acid uptake. Lastly, we found that the transcription levels of *Maoa* and 5‐hydroxyindoleacetic acid (5‐HIAA), a serotonin degradation product, increased in virally infected or poly (I:C)‐treated mice.

## 3 NEUROCOGNITIVE CONSEQUENCES

**FIGURE 2:**
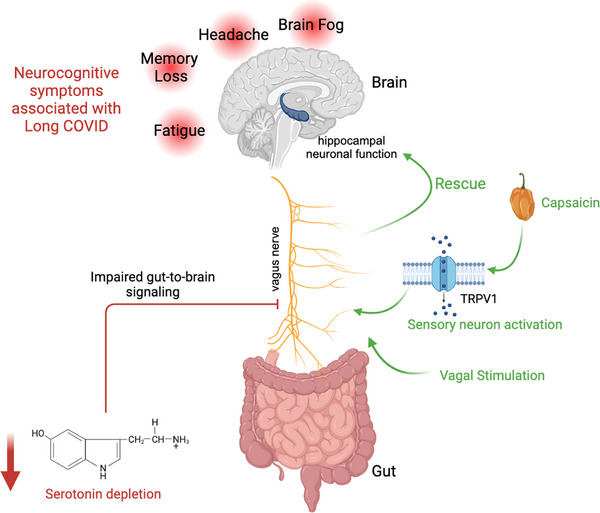
Neurocognitive effects of serotonin depletion and potential therapeutics.

Long COVID is characterised by a wide spectrum of symptoms including fatigue, cognitive difficulties, headaches, endurance loss, sleep disturbances, anxiety and memory lapses. Given the crucial role of serotonin in peripheral and central nervous system function, we sought to explore the physiological consequences of serotonin depletion and its association with neurocognitive symptoms (Figure 2).

Utilising mouse models exposed to acute and chronic infections, we employed the novel object recognition paradigm to assess cognitive ability. We found that infection or poly(I:C) treatment impaired memory in the novel object recognition test. Notably, the selective serotonin reuptake inhibitor (SSRI) fluoxetine restored cognitive function in mice injected with poly(I:C). Furthermore, supplementation of the serotonin precursor 5‐hydroxytryptophan (5‐HTP) not only reinstated peripheral serotonin levels but also demonstrated a reversal of cognitive impairment. None of the peripheral interventions tested in the study altered brain serotonin levels amidst viral inflammation, suggesting a pivotal role of peripheral serotonin reduction in regulating memory function.

How does peripheral serotonin communicate with the brain? We found reduced neuronal activity in the brainstem of poly(I:C)‐treated mice during novelty exposure, suggesting a possible involvement of sensory neurons which terminate in this region of the brain. Furthermore, cognitive impairment induced by viral inflammation was successfully reversed by the administration of capsaicin, a Trpv1 ligand and potent stimulant of sensory neurons. Consistently, when we used chemogenetics to selectively activate Phox2b‐expressing vagal neurons during viral inflammation, we observed a restoration in hippocampal neuron activity and memory function. Further validation came from an experiment using a pharmacological agonist of serotonin receptors on vagal neurons, which emphasised the importance of serotonin signalling through the vagus nerve in regulating cognition.

These results suggest an important role for the vagus nerve, a key mediator of sickness behaviour that has also been implicated in the pathophysiology of chronic fatigue syndrome.^4^, ^5^ However, unravelling the precise circuitry through which sensory neurons relay peripheral inflammation to the brain remains to be explored in future research. Understanding these complexities holds promise for refining therapeutic approaches to address neurocognitive challenges in PASC patients.

## 4 FUTURE OUTLOOK

Beyond SARS‐CoV‐2, low serotonin levels have been documented in other conditions characterised by high levels of type I interferons, such as systemic lupus erythematosus and multiple sclerosis, suggesting broader implications for these findings.^6^, ^7^, ^8^ Our findings suggest that interventions aimed at eliminating the trigger of chronically elevated interferon levels, restoring peripheral serotonin signalling or vagus nerve activation might have clinical utility in the prevention or treatment of Long COVID.

The clinical efficiency of these approaches, however, first requires detailed evaluation. A Dutch study including 95 patients showed a significant reduction in symptoms with SSRIs; however, this was not a randomised controlled trial.^9^ A preprint detailing a multicentre retrospective study of 17 933 patients by the National COVID Cohort Collaborative (N3C) found a 26% reduction in the relative risk of PASC in patients who received an SSRI compared to unexposed controls.^10^ Extensive, double‐blind, prospective studies are needed to establish whether SSRIs or other interventions targeting peripheral serotonin signalling could play a role in managing the long‐term disease burden of COVID‐19. Serotonin precursor supplementation could be an alternative potential therapeutic avenue, demanding rigorous formulation testing for safety and efficacy.

Overall, our study proposes a pathway linking viral persistence, sustained interferon responses, serotonin depletion and vagal dysfunction to neurocognitive symptoms in Long COVID and potentially other postviral syndromes. The findings emphasise the necessity for extensive investigations into sensory neuron dysfunction in PASC. Directly establishing the connection between viral reservoirs in the gastrointestinal tract, sustained inflammatory responses, and PASC manifestations in Long COVID patients remains a significant gap in the field, calling for the initiation of large‐scale studies to investigate this possible molecular etiology underlying postviral conditions.

## AUTHOR CONTRIBUTIONS

SL and ASD performed literature research and wrote the manuscript. ML guided the literature research and edited the manuscript. All authors contributed to the article and approved the submitted version.

## CONFLICT OF INTEREST STATEMENT

The authors declare that they have no conflicts of interest.

## ETHICS STATEMENT

Not applicable
